# Seroprevalence of Herpes Simplex Virus Infection in HIV Coinfected Individuals in Eastern India with Risk Factor Analysis

**DOI:** 10.1155/2015/537939

**Published:** 2015-10-19

**Authors:** Soumyabrata Nag, Soma Sarkar, Debprasad Chattopadhyay, Sanjoy Bhattacharya, Rahul Biswas, Manideepa SenGupta

**Affiliations:** ^1^Department of Microbiology, IIMSAR & BCRH, Haldia, West Bengal, India; ^2^Department of Microbiology, Medical College Kolkata, 88 College Street, Kolkata, West Bengal, India; ^3^ICMR Virus Unit, I.D. and B.G. Hospital, GB-4, 1st Floor, 57 Dr. S. C. Banerjee Road, Beliaghata, Kolkata, India; ^4^Department of Medicine, Medical College Kolkata, 88 College Street, Kolkata, West Bengal, India; ^5^Department of Community Medicine, A.I.I.H. & P.H., Kolkata, West Bengal, India

## Abstract

Herpes simplex virus type 2 (HSV-2) is the cause of most genital herpes while HSV-1 is responsible for orolabial and facial lesions. In immunocompromised individuals, like HIV patients, impaired immunity leads to more frequent symptomatic and asymptomatic HSV infection. Fifty-two blood samples from HIV patients with clinically diagnosed HSV infection were taken as cases, while 45 blood samples each from HIV-infected (HIV control) and noninfected patients without any herpetic lesion (non-HIV control) were taken as control. Serum was tested for IgM and IgG antibodies of both HSV-1 and HSV-2 by ELISA. The seroprevalence was compared among the three groups of study population, considering the demographic and socioeconomic parameters. The HSV-2 IgM was significantly higher (*p* < 0.005) in the HIV patient group (34.6%) than the HIV control (2.2%) and non-HIV control (2.2%) groups, whereas HSV-2 IgG seroprevalence was higher in both HIV patient (61.5%) and HIV control (57.8%) groups than the non-HIV control group (17.8%). The prevalence of HSV-2 was significantly higher in persons with multiple partners and in the reproductive age group. The overall seroprevalence of HSV-1 IgM was too low (<5%), whereas it was too high (about 90%) with HSV-1 IgG in all three study groups.

## 1. Introduction 

Most Herpes simplex virus type 1 (HSV-1) and type 2 (HSV-2) infections are subclinical. However, in symptomatic infections, the clinical manifestations are characterized by recurrent orolabial and facial lesions in HSV-1 and recurrent vesicular, ulcerative genital or anal lesions in HSV-2 [1–3].

HSV is a life-long infection and serological testing provides the best method to estimate its prevalence. Since 1976, the CDC has monitored the HSV-2 seroprevalence in the United States through the National Health and Nutrition Examination Survey (NHANES). Reports indicate that HSV-2 prevalence was increased to 31% between 1976 and 1980 (NHANES II) and was decreased to 21.0% in 1988–1994 (NHANES III) and 17.0% in 1999–2004. In 2005–2008 it was 16.2%, which was statistically same with the seroprevalence in 1999–2004 [4].

Classically, HSV-1 is acquired in childhood through contact, whereas HSV-2 is transmitted sexually. After initial infection, the virus persists for life in a latent form in neurons of the host, periodically reactivate to cause recurrent episodes. Daily suppressive therapy with acyclovir, famciclovir, and valacyclovir decreases HSV shedding dramatically and thereby decreases transmission along with decreased HIV viral loads. Vaccines, interleukins, interferons, therapeutic proteins, antibodies, immunomodulators, small-molecule drugs, and inhibitors of the HSV helicase-primase are in the developmental stages. It is increasingly evident that HSV-2 facilitates HIV transmission [5, 6] which strengthens the importance of the implementation of available HSV control methods [7–9]. The majority of HSV infections are asymptomatic or silent and thus, the infected people shedding the virus are potentially infectious. Therefore, seroepidemiological studies are critical to understand the pattern and distribution of infection within populations [9].

Till date, a limited amount of data on the HSV prevalence and its association with HIV infection are available in Eastern India, particularly in West Bengal. Hence, the aim of this study was to find out the prevalence of HSV infection in HIV patients attending the HIV Clinic of Medical College and Hospital, Kolkata. Specifically, we sought to know the prevalence of HSV-1 and HSV-2 antibodies (both IgM and IgG) in HIV patients with herpetic blister and/or ulcer (HIV group), compared to that in both HIV and non-HIV patients without any herpetic blister and/or ulcer (HIV control) and non-HIV control group. Moreover, the associations, if any, with various demographic, socioeconomic, and behavioral factors were correlated.

## 2. Methods 

After obtaining the institutional ethical clearance, 52 blood samples were collected from patients of both sexes of 18–55 years of age attending the HIV Clinic of Medical College and Hospital, Kolkata, with oral or genital blisters (clinically diagnosed as Herpes simplex lesions) from April 2012 to March 2013. The HIV control group consisted of 45 blood samples, collected from age- and sex-matched HIV seropositive individuals of the clinic, while HIV seronegative blood collected from the Surgery and Gynaecology OPD served as a non-HIV control. Informed consent was obtained from each individual prior to collection of blood. The personal, demographical, and clinical data of all the patients were obtained by a pretest questionnaire containing name, age, sex, socioeconomic status, occupation, marital status, contact history, medical history, sexual behaviour, risk factors, knowledge of STDs (particularly HSV and HIV/AIDS), and clinical symptoms. Patients below 18 years or above 55 years of age, suffering from critical or deteriorating diseases, or with a history of receiving antiviral therapy aside from ART were excluded from the study.

Serum separated from blood samples collected by venipuncture was tested for HSV-1 and HSV-2 (IgG and IgM) antibodies, using commercial ELISA kits (SERION ELISA classic; Manufacturer Fabricant; Institut Virion/Serion GmbH, Germany) that distinguished the type-specific antibody response of both viruses. Microtitre plates of SERION ELISA classic HSV-1 and HSV-2 IgG were coated with recombinant glycoprotein gG1 or gG2, respectively. The use of envelope proteins gG1 in HSV-1 IgG and gG2 in HSV-2 IgG allowed differentiation of type-specific antibody response to HSV-1 and HSV-2. Microtitre plates of SERION ELISA classic HSV-1 IgM and HSV-2 IgM were coated with the corresponding whole virus antigen to ensure immediate and sensitive detection of acute infections. All the tests were done according to the manufacturer's instructions. To fix the cut-off ranges, the mean absorbance value of the supplied standard serum (STD) was multiplied with the numerical data quality control provided by the manufacturer, for example, OD = 0.502 × MW (STD) with upper cut-off and OD = 0.352 × MW (STD) with lower cut-off.

If the measured mean absorbance value of the supplied standard serum is 0.64, the range of the cut-off is in between 0.225 and 0.321.

Statistical analysis was done by a statistician using standard statistical software (SPSS). Data were entered into Microsoft Excel (2007) and further exported to SPSS version 16.0 for analysis. Pearson's chi-square test was performed at 95% confidence interval and significant level was accepted at *p* < 0.05. *p* values were calculated to observe any statistically significant difference among the seroprevalence of HSV-1 and 2 antibodies (IgM and IgG) in different study groups.

## 3. Results 

**Figure 1:**
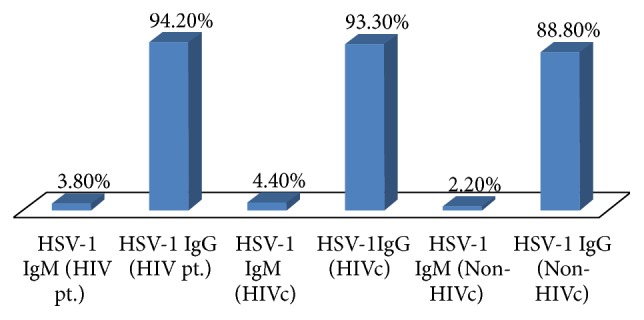
Prevalence of HSV-1 IgM and IgG in HIV pt., HIVc, and non-HIVc group.

**Figure 2:**
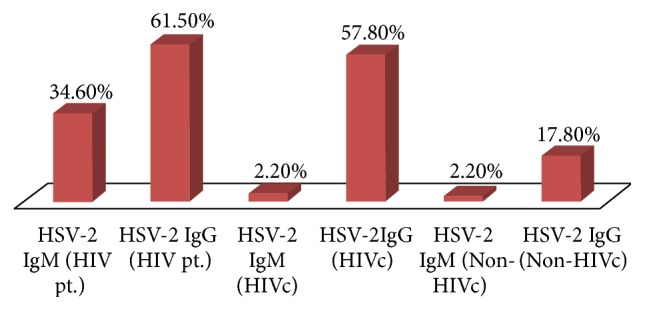
Prevalence of HSV-2 IgM and IgG in HIV pt., HIVc, and non-HIVc group.

Most of the patients participating in the three study groups were males, between 26–45 years, married, literate, and belonged to the upper lower or lower middle class background while most HIV seropositive patients had multiple partners (Table 1). The seroprevalence of HSV-l IgM was found to be very low (<5%), while HSV-l IgG was very high (≈90%) in all three groups (Figures 1 and 2). This suggest a high prevalence of HSV-1 in the general population irrespective of age, sex, literacy, and socioeconomic status (Table 2).

On the other hand, HSV-2 IgM seroprevalence was significantly higher (*p* value < 0.005 by *χ* ^2^ test) in the HIV patient group (34.6%) than the HIV control (2.2%) and non-HIV control (2.2%) group (Tables 3 and 4). In comparison to the non-HIV control group, sera from HIV patients were 23 times more reactive for HSV-2 IgM (odds ratio −23.294; 95% confidence interval 2.961–183.278). Further, the seroprevalence was found to be higher in males of 18–25 years' having more than one partner, literate, and in the upper lower socioeconomic class (Table 3).

HSV-2 IgG seroprevalence was higher in both HIV patient (61.53%) and HIV control (57.78%) groups than the non-HIV control group (17.78%). When compared with the non-HIV control group, the HIV patient group was 29 times (odds ratio −29.421; 95% confidence interval −6.331–136.720) and the HIV control group was 34 times (odds ratio −34.400; 95% confidence interval −7.495–157.895) more likely to be seropositive for HSV-2 IgG, significant at 5% level (*p* value < 0.005). However, HSV-2 IgG did not vary significantly among patients of different age groups, sex, socioeconomic strata, and literacy levels but varied significantly with the number of partners among the patients of the HIV-patient and the non-HIV control group (*p* value < 0.05).

## 4. Discussion 

HSV infection is highly prevalent worldwide and varies between regions and populations.

In this study, it was found that the overall seroprevalence of HSV-2 IgG was 42.3%. While it was 59.79% in HIV-infected patients (61.53% in case and 57.7% in control), and only 17.78% in the non-HIV group. However, higher rates of coinfection with HIV and HSV-2 ranging from 62.7–100% [10–12], 88% and 91% [13] have been reported in the US, Haiti, and Central African Republic, respectively, which was similar to the control group of this study. Another study on hospitalized patients and blood donors in Germany revealed overall 12.8% HSV-2 seropositivity, including 15% females and 10.5% males, but the prevalence in non-HIV control group was 17.78% (20% in males and 15% in females) [14].

There are several possible biological mechanisms where HSV-2 acts as a cofactor in HIV acquisition or transmission. First, the HSV-2 reactivations result in mucosal or epithelial disruption, creating a portal of exit or entry for HIV, to which the activated CD4 cells are recruited [14]. There also appear to be several cellular interactions that promote the establishment of HIV infection and its coinfection with HSV-2 which may lead to the creation of “pseudotypes” (i.e., HSV-2 particles containing the HIV genome enveloped with HSV surface glycoprotein). This allows HSV to infect the cells that could not be infected by HIV alone [11]. The HSV-2 infection may also promote the increased expression of the HIV target cells (i.e., the CCR5+ CD4 cells and the immature dendritic cells) [12].

In our study, the seroprevalence of HSV-1 IgG was found to be very high in all the study groups (overall 92.3%), showing a good correlation with the German study, where the prevalence of HSV-I antibodies showed a steady increase with age and reached high levels (88%) among patients aged 40 years or older [15]. In the German study, the seropositivity of HSV-1 (91.1%) and HSV-2 (47.9%) in HIV-infected populations supports our observation of 93.81% and 59.79% in the present study. However, the higher seropositivity of HSV-2 in males in this study was probably due to limited sample size. Higher prevalence of HSV-1 antibodies (73.3%) among 168 HIV-antibody negative and 132 HIV-antibody positive men, with no difference between HIV seronegative and seropositive men (*p* value = 0.48), while about 20% of HIVseronegative and 61% of seropositive men showed antibodies to HSV-2 (*p* < 0.0001). Similarly, 83.5% and 63.4% seroprevalence of HSV-1 and HSV-2 among patients at higher risk for HIV reported by Lupi [17], is similar to the findings in this study.

The present cross-sectional study on seroprevalence of HSV-2 corroborated the prospective observational study of Patel et al. [18]. Similar results on the seroprevalence of HSV-2 in adult HIV-infected patients and blood donors were also reported by Rode et al. [19] from Croatia. Agabi et al. [20] reported a very high prevalence of HSV-2 (87%) among the patients attending STD Clinic in Jos, Nigeria, which was significantly higher than the present finding, probably due to the differences in sexual behavior and higher prevalence of HIV in Nigeria. Moreover, in this study, genital Herpes (genital blisters: 13, genital ulcers: 35) obtained from 62.3% HSV-2 seropositive HIV subjects indicates that about 37.7% of HIV patients were unaware of their HSV-2 infection.

*Strengths and Limitations*

1. Limited amounts of data are available on the seroprevalence of HSV and its association with HIV infection in Eastern India. Hence, this study was conducted to find out the prevalence of HSV-1 and HSV-2 antibodies (both IgM and IgG) in an HIV patient group, compared with the seroprevalence in an HIV control and non-HIV control group(s) with their demographic, socioeconomic, and behavioural factors.
2. The increased number of HSV seropositivity among HIV positive samples corroborate the fact that there is a synergistic relationship between HIV and HSV infection.
3. Promoting awareness on HSV, its silent epidemic potential, and the role of HSV-2 treatment to decrease HIV transmission and disease progression may have substantial public health benefits.

However, small sample size was the limitation of the study.

## 5. Conclusions 

The HSV seropositivity was found to be higher in HIV positive patient samples (HSV-2 and HSV-l were 59.79 and 93.81%) when compared to non-HIV population (HSV-2 and HSV-l were 17.78 and 88.88%). Thus, it was found that HSV-2 was more common in HIV-infected than in non-HIV-infected individuals. The increased number of HSV seropositivity among HIV positive samples indicates that there is a synergistic relationship between HIV and HSV infection. Moreover, genital herpes (blisters: 13, ulcers: 35) presented by 62.3% of the HSV-2 seropositive HIV subjects indicates that 37.7% of HIV patients were unaware of their HSV-2 infection, suggesting that the awareness of HSV-2 treatment to decrease HIV transmission and disease progression may have substantial public health benefits.

## Figures and Tables

**Table 1:** Distribution of study population according to various parameters.

Overall demographic profile studied	HIV patients with HSV blister/ulcer (HIV patient group)	HIV patients without HSV blister/ulcer (HIV control group)	Non-HIV patients without HSV blister/ulcer (non-HIV control group)
	*n* = 52	*n* = 45	*n* = 45
Age			
18–25 years	8 (15.38%)	8 (17.78%)	9 (20%)
26–35 years	14 (26.92%)	17 (37.78%)	8 (17.78%)
36–45 years	23 (44.23%)	15 (33.33%)	21 (46.67%)
46–55 years	7 (13.46%)	5 (11.11%)	7 (15.55%)
Gender			
Male	30 (57.7%)	23 (51.11%)	25 (55.56%)
Female	22 (42.3%)	22 (48.89%)	20 (44.44%)
Marital status			
Never married	2 (3.85%)	5 (11.11%)	11 (24.44%)
Married	44 (84.62%)	31 (68.89%)	30 (66.67%)
Separated	1 (1.92%)	1 (2.22%)	2 (4.44%)
Widowed	5 (9.61%)	8 (17.78%)	2 (4.44%)
Socioeconomic status			
Lower (L)	4 (7.69%)	3 (6.67%)	2 (4.44%)
Upper lower (UL)	30 (57.69%)	18 (40%)	15 (33.33%)
Lower middle (LM)	17 (32.69%)	18 (40%)	18 (40%)
Upper middle (UM)	1 (1.92%)	3 (6.67%)	7 (15.56%)
Upper (U)	0	3 (6.67%)	3 (6.67%)
Literacy level			
Illiterate (Ill)	7 (13.46%)	11 (24.44%)	8 (17.78%)
Up to primary (P)	29 (55.77%)	3 (6.67%)	4 (8.89%)
Up to middle (M)	9 (17.31%)	17 (37.78%)	10 (22.22%)
Secondary (S)	4 (7.69%)	10 (22.22%)	12 (26.67%)
HS and above (H)	3 (5.77%)	4 (8.89%)	11 (24.44%)
Number of partners			
0	0	9 (20%)	8 (17.78%)
1	28 (53.85%)	9 (20%)	31 (68.89%)
>1	24 (46.15%)	27 (60%)	6 (13.33%)

Different socioeconomic classes as per modified (for 2012) Prasad's Scale of socioeconomic status are based on per capita income in Rupees/month.

Lower = <585; upper lower = 585–1169; lower middle = 1170–1949; upper middle = 1950–3899; upper = ≥3900.

**Table 2:** Seroprevalence of HSV-1 IgM and HSV-1 IgG antibody.

	HIV patient group				HIV control group				Non-HIV control group			
	Number of pts.	HSV-1 IgM		HSV-1 IgG		HSV-1 IgM		HSV-1 IgG		HSV-1 IgM		HSV-1 IgG
Overall	*n* = 52	2 (3.8%)	*n* = 52	49 (94.2%)	*n* = 45	2 (4.4%)	*n* = 45	42 (93.3%)	*n* = 45	1 (2.2%)		40 (88.9%)

According to gender												
Male	*n* = 30	3.3%	*n* = 30	93.3%	*n* = 23	4.3%	*n* = 23	95.7%	*n* = 25	4%	*n* = 25	92%
Female	*n* = 22	4.5%	*n* = 22	95.5%	*n* = 22	4.5%	*n* = 22	90.0%	*n* = 20	0	*n* = 20	85%

According to age (in yrs)												
18–25	*n* = 8	12.5%	*n* = 8	100%	*n* = 8	0	*n* = 8	100%	*n* = 9	0	*n* = 9	100%
26–35	*n* = 14	0	*n* = 14	100%	*n* = 17	5.9%	*n* = 17	94.1%	*n* = 8	0	*n* = 8	87.5%
36–45	*n* = 23	4.3%	*n* = 23	87%	*n* = 15	6.7%	*n* = 15	86.7%	*n* = 21	4.8%	*n* = 21	85.7%
46–55	*n* = 7	0	*n* = 7	100%	*n* = 5	0	*n* = 5	100%	*n* = 7	0	*n* = 7	85.7%

According to number of partners												
0	*n* = 0	0	*n* = 0	0	*n* = 9	0	*n* = 9	100%	*n* = 8	0	*n* = 8	7.5%
1	*n* = 28	0	*n* = 28	100%	*n* = 9	0	*n* = 9	7.7%	*n* = 31	3.3%	*n* = 31	90.3%
>1	*n* = 24	8.3%	*n* = 24	87.5%	*n* = 27	7.4%	*n* = 27	9.6%	*n* = 6	0	*n* = 6	100%

According to Income groups												
L	*n* = 4	25%	*n* = 4	75%	*n* = 3	0	*n* = 3	100%	*n* = 2	0	*n* = 2	100%
UL	*n* = 30	3.3%	*n* = 30	93.3%	*n* = 18	5.5%	*n* = 18	100%	*n* = 15	0	*n* = 15	93.3%
LM	*n* = 17	0	*n* = 17	100%	*n* = 18	5.5%	*n* = 18	88.9%	*n* = 18	5.5%	*n* = 18	88.9%
UM	*n* = 1	0	*n* = 1	100%	*n* = 3	0	*n* = 3	100%	*n* = 7	0	*n* = 7	71.4%
U	*n* = 0	0	*n* = 0	0	*n* = 3	0	*n* = 3	66.7%	*n* = 3	0	*n* = 3	100%

According to literacy status												
Ill	*n* = 7	0	*n* = 7	100%	*n* = 11	9.1%	*n* = 11	81.8%	*n* = 8	0	*n* = 8	100%
P	*n* = 29	3.4%	*n* = 29	93.1%	*n* = 3	33.3%	*n* = 3	100%	*n* = 4	0	*n* = 4	100%
M	*n* = 9	11.1%	*n* = 9	100%	*n* = 17	0	*n* = 17	100%	*n* = 10	0	*n* = 10	90%
S	*n* = 4	0	*n* = 4	75%	*n* = 10	0	*n* = 10	100%	*n* = 12	8.3%	*n* = 12	83.3%
H	*n* = 3	0	*n* = 3	100%	*n* = 4	0	*n* = 4	75%	*n* = 11	0	*n* = 11	81.8%

L, lower; UL, upper lower; LM, lower middle; UM, upper middle; U, upper; Ill, illiterates; P, primary; M, middle; S, secondary; H, HS and above.

**Table 3:** Seroprevalence of HSV-2 IgM and HSV-2 IgG antibody.

	HIV patient group				HIV control group				Non-HIV control group			
	Number of pts.	HSV-2 IgM		HSV-2 IgG		HSV-2 IgM		HSV-2 IgG		HSV-2 IgM		HSV-2 IgG
Overall	*n* = 52	18 (34.6%)	*n* = 52	32 (61.5%)	*n* = 45	1 (2.2%)	*n* = 45	26 (57.8%)	*n* = 45	1 (2.2%)	*n* = 45	8 (17.8%)

According to gender												
Male	*n* = 30	40%	*n* = 30	63.3%	*n* = 23	0	*n* = 23	60.9%	*n* = 25	4%	*n* = 25	20%
Female	*n* = 22	27%	*n* = 22	59.1%	*n* = 22	4.5%	*n* = 22	54.5%	*n* = 20	0	*n* = 20	15%

According to age (in yrs)												
18–25	*n* = 8	25%	*n* = 8	62.5%	*n* = 8	0	*n* = 8	75%	*n* = 9	11.1%	*n* = 9	11.1%
26–35	*n* = 14	35.7%	*n* = 14	85.7%	*n* = 17	5.9%	*n* = 17	47.1%	*n* = 8	0	*n* = 8	12.5%
36–45	*n* = 23	34.8%	*n* = 23	52.2%	*n* = 15	0	*n* = 15	60%	*n* = 21	0	*n* = 21	23.8%
46–55	*n* = 7	42.9%	*n* = 7	42.9%	*n* = 5	0	*n* = 5	60%	*n* = 7	0	*n* = 7	14.3%

According to number of partners												
0	*n* = 0	0	*n* = 0	0	*n* = 9	0	*n* = 9	55.5%	*n* = 8	0	*n* = 8	0
1	*n* = 28	35.7%	*n* = 28	42.8%	*n* = 9	0	*n* = 9	55.5%	*n* = 31	0	*n* = 31	12.9%
>1	*n* = 24	33.3%	*n* = 24	83.3%	*n* = 27	3.7%	*n* = 27	59.2%	*n* = 6	16.6%	*n* = 6	66.7%

According to income groups												
L	*n* = 4	25%	*n* = 4	75%	*n* = 3	0	*n* = 3	66.7%	*n* = 2	0	*n* = 2	0
UL	*n* = 30	43.3%	*n* = 30	63.3%	*n* = 18	0	*n* = 18	61.1%	*n* = 15	0	*n* = 15	26.7%
LM	*n* = 17	23.5%	*n* = 17	58.8%	*n* = 18	0	*n* = 18	55.5%	*n* = 18	5.5%	*n* = 18	16.7%
UM	*n* = 1	0	*n* = 1	0	*n* = 3	33.3%	*n* = 3	0	*n* = 7	0	*n* = 7	14.2%
U	*n* = 0	0	*n* = 0	0	*n* = 3	0	*n* = 3	100%	*n* = 3	0	*n* = 3	0

According to literacy status												
Ill	*n* = 7	28.5%	*n* = 7	71.4%	*n* = 11	0	*n* = 11	45.4%	*n* = 8	0	*n* = 8	25%
P	*n* = 29	41.3%	*n* = 29	55.1%	*n* = 3	0	*n* = 3	66.7%	*n* = 4	0	*n* = 4	25%
M	*n* = 9	11.1%	*n* = 9	66.7%	*n* = 17	0	*n* = 17	58.7%	*n* = 10	0	*n* = 10	20%
S	*n* = 4	50%	*n* = 4	100%	*n* = 10	10%	*n* = 10	70%	*n* = 12	8.3%	*n* = 12	16.6%
H	*n* = 3	33.3%	*n* = 3	33.3%	*n* = 4	0	*n* = 4	50%	*n* = 11	0	*n* = 11	9.1%

L, lower; UL, upper lower; LM, lower middle; UM, upper middle; U, upper; Ill, illiterates; P, primary; M, middle; S, secondary; H, HS and above.

**Table 4:** Seroprevalence of HSV-1 and 2 antibodies (IgM and IgG) in different study groups.

	HIV patient group			HIV control group			Non-HIV control group			Total			Chi square test (*p* value)
	R	NR	T	R	NR	T	R	NR	T	R	NR	T	
HSV1 IgM	**2 (3.8%)**	**50 (96.2%)**	**52 (100%)**	**2 (4.4%)**	**43 (95.6%)**	**45 (100%)**	**1 (2.2%)**	**44 (97.8%)**	**45 (100%)**	**5 (3.5%)**	**137 (96.5%)**	**142 (100%)**	0.838 (not statistically significant)
HSV1 IgG	**49 ** **(94.2%)**	**3 (5.8%)**	**52 (100%)**	**42 ** **(93.3%)**	**3 (6.7%)**	**45 (100%)**	**40 ** **(88.9%)**	**5 ** **(11.1%)**	**45 (100%)**	**131 ** **(92.3%)**	**11 ** **(7.7%)**	**142 ** **(100%)**	0.585 (not statistically significant)
HSV2 IgM	**18 ** **(34.6%)**	**34 ** **(65.4%)**	**52 **(100%)	**1 ** **(2.2%)**	**44 ** **(97.8%)**	**45 **(100%)	**1 ** **(2.2%)**	**44 ** **(97.8%)**	**45 **(100%)	**20 ** **(14.1%)**	**122 ** **(85.9%)**	**142 (100%)**	**0.000** (statistically significant)
HSV2 IgG	**32 **(61.5%)	**20 **(38.5%)	**52 **(100%)	**26 **(57.8%)	**19 **(42.2%)	**45 **(100%)	**8 **(17.78%)	**37 **(82.22%)	**45 **(100%)	**66 ** **(46.48%)**	**76 ** **(53.52%)**	**142 (100%)**	**0.000 **(statistically significant)
